# Astrocytes with TDP-43 inclusions exhibit reduced noradrenergic cAMP and Ca^2+^ signaling and dysregulated cell metabolism

**DOI:** 10.1038/s41598-020-62864-5

**Published:** 2020-04-07

**Authors:** Jelena Velebit, Anemari Horvat, Tina Smolič, Sonja Prpar Mihevc, Boris Rogelj, Robert Zorec, Nina Vardjan

**Affiliations:** 1 Laboratory of Cell Engineering, Celica Biomedical, 1000 Ljubljana, Slovenia; 2 Laboratory of Neuroendocrinology – Molecular Cell Physiology, Institute of Pathophysiology, Faculty of Medicine, University of Ljubljana, 1000 Ljubljana, Slovenia; 3 Department of Biotechnology, Jožef Stefan Institute, 1000 Ljubljana, Slovenia; 4 Biomedical Research Institute BRIS, 1000 Ljubljana, Slovenia; 5 Faculty of Chemistry and Chemical Technology, University of Ljubljana, 1000 Ljubljana, Slovenia

**Keywords:** Mechanisms of disease, Cellular neuroscience, Amyotrophic lateral sclerosis, Astrocyte, Amyotrophic lateral sclerosis

## Abstract

Most cases of amyotrophic lateral sclerosis (ALS) and frontotemporal dementia (FTD) have cytoplasmic inclusions of TAR DNA-binding protein 43 (TDP-43) in neurons and non-neuronal cells, including astrocytes, which metabolically support neurons with nutrients. Neuronal metabolism largely depends on the activation of the noradrenergic system releasing noradrenaline. Activation of astroglial adrenergic receptors with noradrenaline triggers cAMP and Ca^2+^ signaling and augments aerobic glycolysis with production of lactate, an important neuronal energy fuel. Astrocytes with cytoplasmic TDP-43 inclusions can cause motor neuron death, however, whether astroglial metabolism and metabolic support of neurons is altered in astrocytes with TDP-43 inclusions, is unclear. We measured lipid droplet and glucose metabolisms in astrocytes expressing the inclusion-forming C-terminal fragment of TDP-43 or the wild-type TDP-43 using fluorescent dyes or genetically encoded nanosensors. Astrocytes with TDP-43 inclusions exhibited a 3-fold increase in the accumulation of lipid droplets versus astrocytes expressing wild-type TDP-43, indicating altered lipid droplet metabolism. In these cells the noradrenaline-triggered increases in intracellular cAMP and Ca^2+^ levels were reduced by 35% and 31%, respectively, likely due to the downregulation of β_2_-adrenergic receptors. Although noradrenaline triggered a similar increase in intracellular lactate levels in astrocytes with and without TDP-43 inclusions, the probability of activating aerobic glycolysis was facilitated by 1.6-fold in astrocytes with TDP-43 inclusions and lactate MCT1 transporters were downregulated. Thus, while in astrocytes with TDP-43 inclusions noradrenergic signaling is reduced, aerobic glycolysis and lipid droplet accumulation are facilitated, suggesting dysregulated astroglial metabolism and metabolic support of neurons in TDP-43-associated ALS and FTD.

## Introduction

Amyotrophic lateral sclerosis (ALS) is a neurodegenerative disorder characterized by the loss of both upper and lower motor neurons in the brain and spinal cord, and by the progressive paralysis of voluntary muscles and death^1,2^. The pathologic hallmark of ALS are cytoplasmic inclusions in motor neurons. In most cases (~95%) of sporadic and familial ALS, TAR DNA-binding protein 43 (TDP-43), encoded by the *TARDBP* gene, has been identified as the key component of these inclusions^1–9^. Moreover, TDP-43 has also been identified as the major protein in inclusions in frontotemporal dementia with ubiquitin-positive inclusions (FTD-U)^2,6^.

TDP-43 is a highly conserved protein (414 amino acids), ubiquitously expressed in all tissues and under physiological conditions, primarily localized to the nucleus; however, low levels are also present in the cytoplasm^2,3,8,10–13^. TDP-43, an RNA-binding protein, is implicated in multiple aspects of RNA processing, including regulation of transcription, splicing, transport, and stabilization of mRNAs. It also regulates microRNA biogenesis and interacts with DNA. Therefore, its perturbance may lead to significant changes in the transcriptome and proteome^14–17^. It consists of an N-terminal domain, two RNA recognition motifs and a C-terminal prion-like glycine-rich domain that mediates protein-protein interactions with other heterogeneous ribonucleoprotein (hnRNP) family members^2,3,11^.

In most pathologic cases, TDP-43 is hyperphosphorylated and ubiquitinated^18^. Although ubiquitination targets TDP-43 aggregates for degradation, TDP-43 begins to accumulate in the cytoplasm, suggesting that additional perturbance in either the ubiquitin-proteasome system or the autophagy pathway can facilitate the accumulation of TDP-43 in ALS and FTD-U^19^. 25-kDa C-terminal fragments of TDP-43 (TDP-43^208–414^) are commonly detected in ALS and FTD-U pathologic specimens, especially in the cerebral cortex, and generation of these fragments is sufficient to initiate a number of events that mirror TDP-43 proteinopathies^2,3,20^.

TDP-43-containing cytoplasmic inclusions are not restricted to motor neurons but are also found in non-neuronal cells, in particular in astrocytes^21^. Astrocytes with TDP-43 inclusions are sufficient to cause motor neuron death in animal models^22,23^ and exhibit autocytotoxicity^7^. Thus, astrocytes were recently proposed to play an active role in controlling ALS disease progression and may even be the primary driver of TDP-43 proteinopathies^2,7^.

Astrocytes are an abundant and heterogeneous subtype of neuroglia in the central nervous system (CNS)^24^, regulating CNS metabolism^25^. With their numerous processes, they are in tight contact with neurons, including motor neurons, and blood vessels. They transport nutrients from the blood stream to neurons and store blood-derived glucose in the form of glycogen as the CNS fuel reserve^26^ and perhaps also as free glucose in endoplasmic reticulum^27^. Astrocytes are considered an important cellular target of noradrenaline (NA), released from the *locus coeruleus* (LC) noradrenergic neurons, which regulates CNS energy metabolism^28–32^. NA binds to G-protein-coupled adrenergic receptors (ARs; α_1_-, α_2_- and β-ARs [β_1_, β_2_, β_3_]) on the surface of brain cells, including astrocytes^33,34^, where ARs are abundantly expressed^35^, changing the intracellular concentration of cyclic adenosine monophosphate ([cAMP]_i_) and Ca^2+^ ([Ca^2+^]_i_)^36–39^. This activates astroglial metabolism, which is mainly controlled by β-AR/cAMP signaling, enhancing glucose uptake, glycogenolysis, aerobic glycolysis, and lactate production^40^. Lactate is considered to be then shuttled to neurons where it is used as fuel by being transformed to pyruvate and entering oxidative phosphorylation^41,42^.

*In vitro* and *in vivo* studies using ALS model systems of superoxide dismutase 1 (SOD1)-related familial ALS have provided first evidence of metabolic dysfunction in ALS astrocytes, particularly in the transporter responsible for the efflux of lactate^43^, dysfunctional astrocytic mitochondria^44^, and lower levels of intracellular lactate upon exposure of astrocytes to glutamate^45^. Besides glucose metabolism, lipid metabolism might also be impaired in neurons and astrocytes during neurodegeneration as observed in animal models^46–48^. If reactive oxygen species (ROS) are elevated in the brain like during neurodegeneration^46^, the glia-neuron lactate shuttle is believed to promote lipid synthesis in neurons. Peroxidized and potentially cytotoxic lipids are then shuttled via apolipoproteins (APO) E and D and fatty acid transport proteins, from neurons to glial cells, causing accumulation of lipid droplets (LDs) in glial cells^47^, dynamic organelles, undergoing regulated, stress-induced biogenesis and/or degradation^49,50^, that play an important role in intracellular lipid metabolism and storage^51^. Consistent with the observations in animal models, a reduced glucose uptake and increased glycogen storage have been observed in the CNS of patients with ALS^52^; both processes highly depend on astrocytes, suggesting alterations in astroglial metabolism. Whether TDP-43 inclusions, a hallmark of ALS and FTD-U pathologies, can in astrocytes *per se* alter astroglial glucose and lipid droplet metabolisms is not known, and was the subject of this study.

The results revealed that overexpression of the C-terminal fragment of TDP-43 (amino acids 208–414) results in cytoplasmic TDP-43 inclusions in cultured rat cortical astrocytes, typical for ALS and FTD-U pathologies. These cells accumulated LDs. To measure the effect of TDP-43 inclusions in single cells on the dynamics of AR-induced Ca^2+^, cAMP signaling and lactate production in astrocytes, we used real-time fluorescence microscopy, Ca^2+^ indicator Fluo-4 AM dye, and genetically encoded FRET (fluorescence resonance energy transfer)-based cAMP (Epac1-camps^53^) and lactate (Laconic^54^) nanosensors. NA induced increases in [cAMP]_i_, [Ca^2+^]_i_, and intracellular concentration of lactate ([lactate]_i_) in astrocytes expressing wild-type (WT) TDP-43 and in astrocytes with cytoplasmic TDP-43 inclusions (expressing C-terminal fragment of TDP-43). The amplitude and the time constant of the increase in [cAMP]_i_ were, however, reduced by 35% and 50%, respectively, and the peak amplitude and the cumulative [Ca^2+^]_i_ increase were reduced by 20% and 30%, respectively, in astrocytes with cytoplasmic TDP-43 inclusions (expressing C-terminal fragment of TDP-43) versus astrocytes transfected with WT TDP-43. The amplitude and the rate of increase in [lactate]_i_ upon NA treatment were similar in WT TDP-43 expressing astrocytes and in astrocytes with TDP-43 inclusions, however the responsiveness of astrocytes containing cytoplasmic TDP-43 inclusions to NA with production of lactate was increased by 1.6-fold versus WT TDP-43 expressing cells. Astrocytes with TDP-43 inclusions exhibited a reduced expression of β_2_-ARs and MCT1 transporters, as determined with immunocytochemistry, consistent with reduced cAMP and Ca^2+^ signaling and suggesting decreased astroglial lactate release capacity. Thus, astroglial aerobic glycolysis is altered in astrocytes with cytoplasmic TDP-43 inclusions, which may affect astroglial metabolic support of neurons in ALS and FTD-U. Moreover, these cells accumulated LDs.

## Results

### Overexpression of TDP-43^208–414^ in astrocytes causes cytoplasmic inclusions and lowers the amount of endogenous nuclear TDP-43

**Figure 1:**
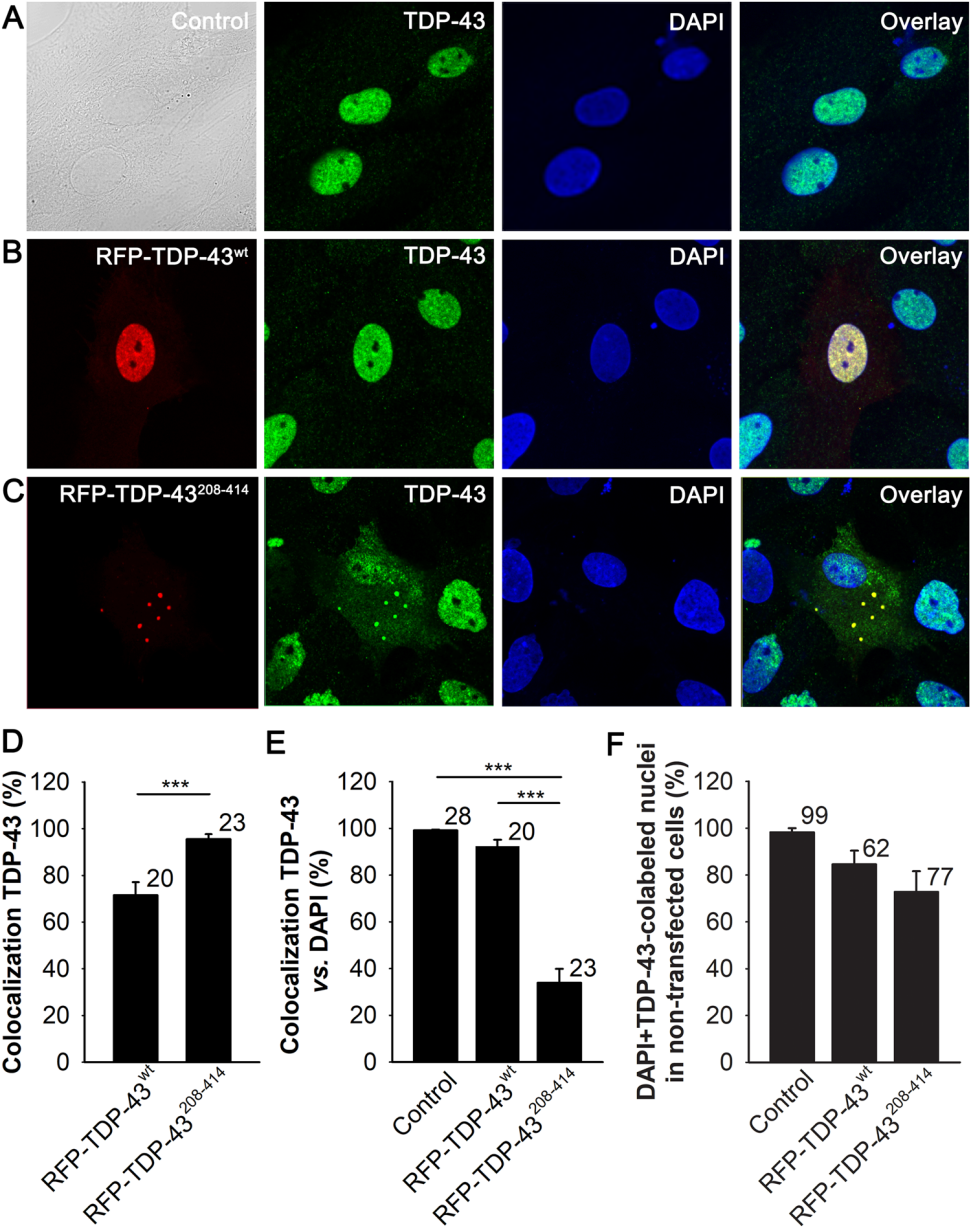
Nuclear RFP-TDP-43^wt^ and cytoplasmic RFP-TDP-43^208–414^ expression in cultured cortical rat astrocytes. (**A**–**C**) Representative fluorescence images of astrocytes immunostained with antibodies against endogenous TDP-43 (green) and labelled with DAPI (blue) in (**A**) control (non-transfected), (**B**) RFP-tagged wild-type TDP-43-expressing astrocytes (RFP-TDP-43^wt^; red) and (**C**) RFP-tagged C-terminal fragment of TDP-43-expressing astrocytes (RFP-TDP-43^208–414^; red). Note the red fluorescent inclusions in the cytoplasm of astrocytes expressing RFP-TDP-43^208–414^ and nuclear expression of RFP-TDP-43^wt^. Scale bar, 20 µm. (**D**) Colocalization (%) between RFP fluorescence signal (red) and Alexa Fluor^488^-labelled TDP-43 antibody (green) in astrocytes transfected with RFP-TDP-43^wt^ or RFP-TDP-43^208–414^. (**E**) Colocalization (%) between the total cellular TDP-43 antibody signal and the nuclear DAPI stain in control cells and in cells transfected with RFP-TDP-43^wt^ or RFP-TDP-43^208–414^. Note the low percentage of TDP-43 antibody colocalization with the nuclear DAPI stain in RFP-TDP-43^208–414^-expressing astrocytes, indicating that in these cells, most of the TDP-43 protein resides in the cytoplasm. (**F)** The percentage (%) of DAPI and TDP-43-colabeled nuclei in non-transfected astrocytes (no visible RFP signal) in control experimental group (Control; n = 99 cells from 28 images) and in non-transfected astrocytes around the astrocytes expressing the RFP-constructs in RFP-TDP-43^wt^ (n = 62 cells from 18 images), and RFP-TDP-43^208–414^ (n = 77 cells from 19 images) experimental groups. The numbers by the error bars indicate the number of cells (D, E) or number of cell nuclei (F) analysed. Data are presented as means ± SEM and acquired from at least two different animals. Each experiment (*i*.*e*. coverslip) was performed in duplicate, multiple cells were recorded per coverslip. ***P ≤ 0.001, Mann-Whitney U test (D); ***P ≤ 0.001, Kruskal-Wallis one-way ANOVA on ranks, followed by Dunn’s test (E).

To study how ALS- and FTD-U-associated TDP-43 inclusions in astrocytes affect cell metabolism, in particularly LD and glucose metabolisms, we transfected primary cortical rat astrocytes with the pDNA encoding RFP-tagged C-terminal fragment of TDP-43 (RFP-TDP-43^208–414^), known to generate cytoplasmic inclusions in other cell types^20^, or with the pDNA encoding RFP-tagged WT TDP-43 (RFP-TDP-43^wt^). Using confocal microscopy, we observed that in astrocytes expressing the RFP-TDP-43^wt^ construct, the RFP signal is visible mainly in the cell nucleus (Fig. 1B), whereas in RFP-TDP-43^208–414^-expressing astrocytes, red fluorescent inclusions, typical for ALS and FTD-U pathologies, are observed in the cytoplasm (Fig. 1C). To confirm the subcellular localization of the TDP-43 in cultured rat astrocytes after transfection, we immunostained non-transfected (control) cells and cells expressing RFP-TDP-43^wt^ and RFP-TDP-43^208–414^ with antibodies against TDP-43 (Fig. 1). We observed that the red fluorescent regions represent the RFP-tagged TDP-43-containing subcellular compartments, because they largely colocalized with the cell areas labelled with green Alexa Fluor^488^-labelled TDP-43 antibodies. The degree of colocalization (%) with anti-TDP-43 was 71.5 ± 5.5% (n = 20) and 95.5 ± 2.1% (n = 23) in RFP-TDP-43^wt^- and RFP-TDP-43^208–414^-expressing astrocytes, respectively (Fig. 1D). Since the expression of RFP-TDP-43^wt^ is disperse and RFP-TDP-43^208–414^ is concentrated in inclusions, this complicates the quantitation of expression level of the constructs at cellular level by means of measuring RFP intensity fluorescence signal per total cell area.

Previous studies on different cell types have demonstrated that TDP-43 cytoplasmic inclusions cause nuclear clearance of endogenous TDP-43^5,10,55^. Therefore, we next investigated whether expression of the C-terminal fragment of TDP-43 (TDP-43^208–414^) affects the nuclear levels of endogenous TDP-43 in cultured rat astrocytes. To evaluate this, we performed colocalization analysis between Alexa^488^-labelled TDP-43 antibody and nuclear marker DAPI in control, non-transfected astrocytes, and in astrocytes expressing the RFP-tagged TDP-43 pDNA constructs. The degree of colocalization (%) with DAPI was 99.1 ± 0.4% (n = 28), 92.2 ± 2.9% (n = 20), and 33.9 ± 6.0% (n = 23) in control, RFP-TDP-43^wt^- and RFP-TDP-43^208–414^-expressing astrocytes, respectively (Fig. 1E). The relatively low percentage of TDP-43 antibody colocalization with DAPI in astrocytes expressing the RFP-TDP-43^208–414^, compared with control, non-transfected and RFP-TDP-43^wt^-expressing astrocytes (*P* < 0.001), suggests that the C-terminal fragment of TDP-43 causes partial depletion of endogenous nuclear TDP-43. When we analyzed the extent of DAPI and TDP-43-colabeled nuclei of non-transfected astrocytes (with no visible RFP signal), positioned around astrocytes expressing the RFP-TDP-43 constructs (with visible RFP signal), we observed a small, statistically insignificant reduction trend in percentage of DAPI and TDP-43-colabeled nuclei in the RFP-TDP-43^208–414^ experimental group compared to RFP-TDP-43^wt^ and control experimental group (Fig. 1F). This suggests that the transfection with the RFP-tagged TDP-43^208–414^ construct may affect the endogenous level of nuclear TDP-43 and/or change the antibody staining in some neighboring non-transfected astrocytes.

Taken together, these results demonstrate that in cultured rat astrocytes, expression of the ALS- and FTD-U-linked C-terminal fragment of TDP-43 causes the formation of TDP-43-containing cytoplasmic inclusions and partial sequestration of endogenous nuclear TDP-43 to the cytoplasm.

### Lipid droplet content is increased in the cytoplasm of TDP-43^208–414^-expressing astrocytes

**Figure 2:**
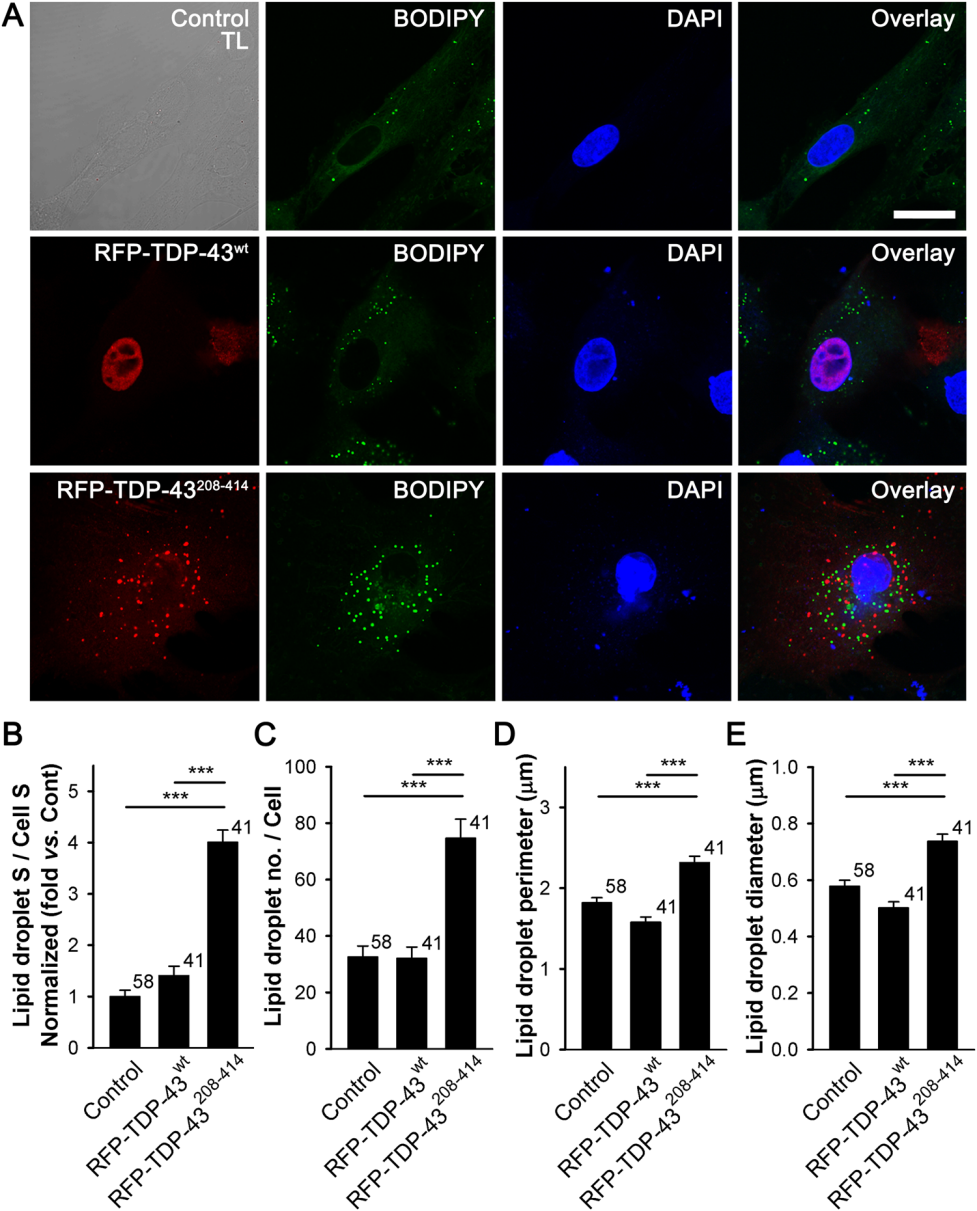
Astrocytes expressing RFP-TDP-43^208–414^ have an increased lipid droplet content compared with RFP-TDP-43^wt^-expressing astrocytes. (**A)** Representative fluorescence images of non-transfected astrocytes (control; upper panels) and astrocytes transfected with RFP-tagged TDP-43^wt^ (middle panels) or TDP-43^208–414^ (lower panels) plasmids (red) and stained with fluorescent lipid droplet (LD) marker BODIPY^493/503^ (BODIPY; green). BODIPY^493/503^ staining was performed 25 h after transfection with pDNA constructs. Nuclei are labelled with DAPI (blue). TL; transmission light. Scale bar, 20 μm. (**B**–**E**) Histogram of BODIPY^493/503^-positive cell cross-sectional area (lipid droplet S; i.e. number of green fluorescence pixels with fluorescence intensity above the threshold of 20% of maximal fluorescence) versus total cell cross-sectional area (cell S; i.e. number of all pixels; **B**), average LD number per cell (**C**), LD perimeter (**D**), and LD diameter (**E**) in control, RFP-TDP-43^wt^- and RFP-TDP-43^208–414^-expressing cells. Numbers adjacent to the error bars indicate the number of cells analysed. Data are presented as means ± SEM and acquired from at least two different animals. Each experiment (*i*.*e*. coverslip) was performed in duplicate, multiple cells were recorded per coverslip. Asterisks denote statistically significant differences (***P < 0.001, Kruskal-Wallis one-way ANOVA on ranks, followed by Dunn’s test).

To evaluate whether the expression of the ALS- and FTD-U-linked C-terminal fragment of TDP-43 affects accumulation of astroglial LDs, we stained non-transfected cells and cells expressing RFP-TDP-43^wt^ and RFP-TDP-43^208–414^ with a fluorescent LD marker BODIPY^493/503^ (Fig. 2). The expression of RFP-TDP-43^208–414^ (n = 41) in astrocytes increased the amount of BODIPY^493/503^-positive cell cross-sectional area, indicating LD content increase, by ~4- and ~3-fold compared with control non-transfected (n = 58) and RFP-TDP-43^wt^-expressing astrocytes, respectively (n = 41; Fig. 2B; *P* < 0.001). Moreover, the average number of LDs per cell (32.0 ± 4.1 (RFP-TDP-43^wt^) versus 74.7 ± 6.7 (RFP-TDP-43^208–414^), *P* < 0.001), LD perimeter (1.6 ± 0.1 μm (RFP-TDP-43^wt^) versus 2.3 ± 0.1 μm (RFP-TDP-43^208–414^), *P* < 0.001), and LD diameter (0.50 ± 0.02 μm (RFP-TDP-43^wt^) versus 0.74 ± 0.03 μm (RFP-TDP-43^208–414^), *P* < 0.001), increased in astrocytes with TDP-43 inclusions (Fig. 2C–E), indicating an increase in the accumulation of LDs.

A small amount of TDP-43 inclusions in some cells can be seen inside the DAPI-labelled nuclear area, thus the role of nuclear TDP-43 inclusions on the accumulation of LDs cannot be excluded. However, since the thickness of the optical section of an individual confocal image was relatively large (1.2 μm) enabling the nuclear and cytosolic fluorescence signals to overlap, it is more likely, that the TDP-43 inclusions seen at the DAPI-labeled nuclear area are in fact located in the cytosol.

### Noradrenaline-mediated increases in [cAMP]_i_ and [Ca^2+^]_i_ are reduced in TDP-43^208–414^-expressing astrocytes

**Figure 3:**
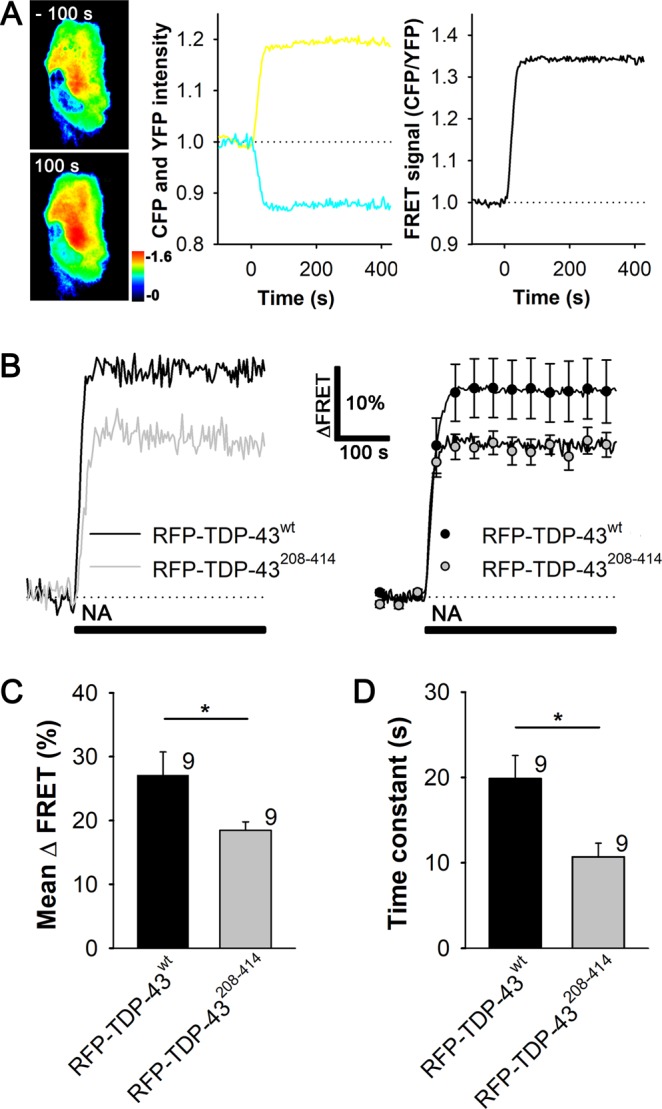
Astrocytes expressing RFP-TDP-43^208–414^ exhibit reduced increase in [cAMP]_i_ upon noradrenaline stimulation compared with RFP-TDP-43^wt^-expressing astrocytes. (**A)** Left panel: pseudocolor FRET (CFP/YFP) signal images of an astrocyte expressing Epac1-camps before (−100 s) and after (100 s) the addition of 100 µM noradrenaline (NA) at *t* = 0. The corresponding pseudocolor scale bar depicts the CFP/YFP values. Middle panel: time courses of the CFP and YFP fluorescence intensities; right panel, Epac1-camps inverse FRET signal (CFP/YFP) normalized to the baseline values upon NA stimulation for the cell shown in (**A**, left panel). Note that the addition of NA leads to an increase in the FRET signal, indicating an increase in [cAMP]_i_. (**B)** Representative (left) and average (right) time-dependent changes in the Epac1-camps FRET signal increase (∆FRET) after the addition of 100 µM NA (black lines) in astrocytes co-expressing RFP-TDP-43^wt^ (black line/circles; n = 9) or RFP-TDP-43^208–414^ (grey line/circles; n = 9). Data are expressed as percentages of the inverse FRET signal (CFP/YFP) relative to the baseline FRET signal. Each data point in the right panel represents the mean ± SEM. (**C**,**D)** Mean changes in the Epac1-camps FRET signal (mean ∆FRET; **C**) and the mean time constants (**D**) after the addition of NA, determined by fitting the exponential functions to the increase in the FRET signal of individual recordings for RFP-TDP-43^wt^ and RFP-TDP-43^208–414^ experimental groups. Numbers adjacent to the error bars depict the number of cells analysed. Data are presented as means ± SEM and acquired from at least two different animals (one cell was recorded per coverslip). Asterisk denotes statistical significance, *P < 0.05, determined by the Student’s t-test.

**Figure 4:**
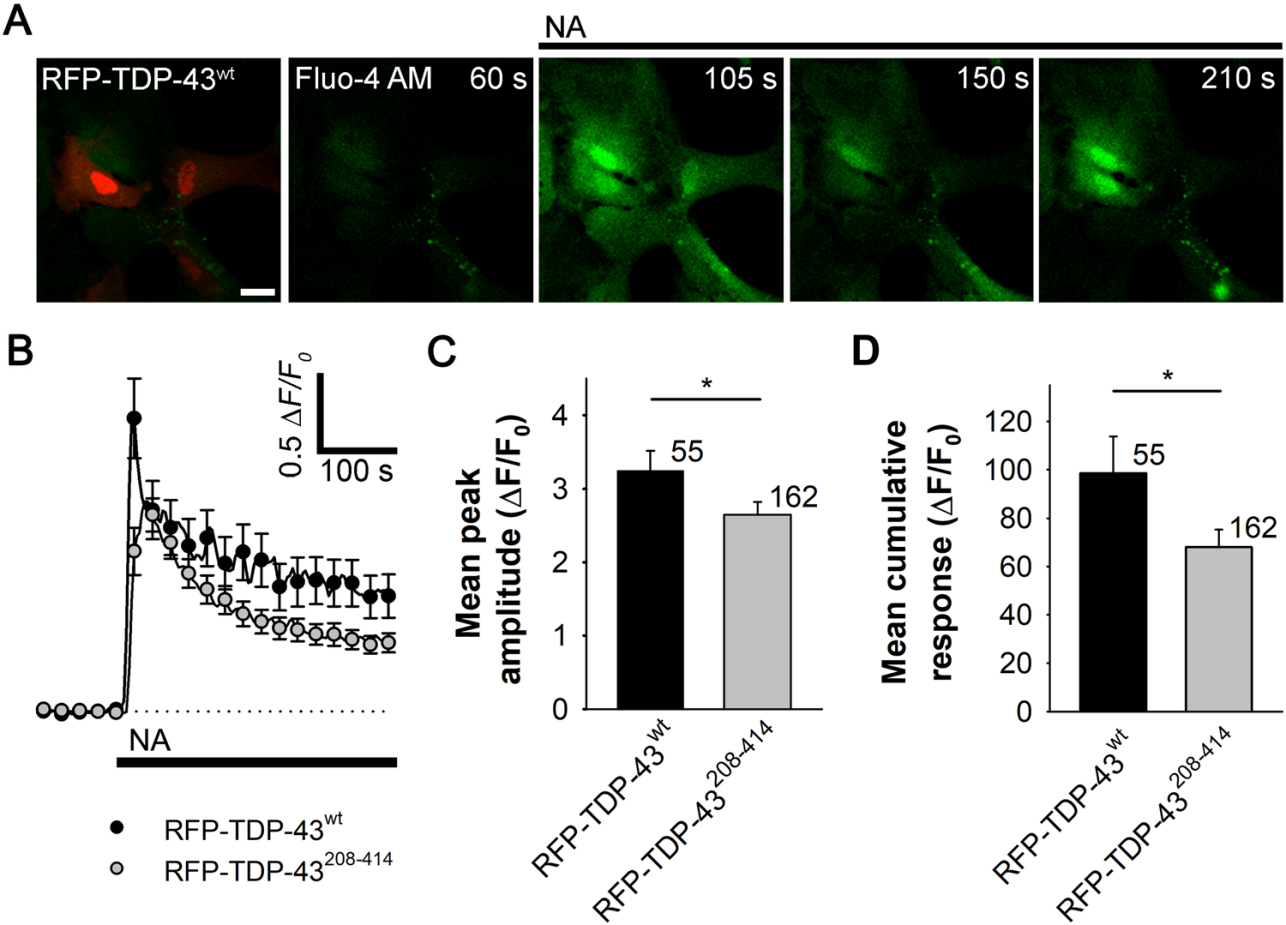
Noradrenaline-mediated Ca^2+^ response is reduced in astrocytes expressing RFP-TDP-43^208–414^ compared with RFP-TDP-43^wt^-expressing astrocytes. (**A)** Representative fluorescence images of RFP-TDP-43^wt^-expressing astrocytes labeled with Ca^2+^ indicator Fluo-4 AM and stimulated with noradrenaline (NA, black line). Scale bar: 20 µm. (**B)** Mean intensity changes in intracellular Ca^2+^ (ΔF/F_0_) upon stimulation with 100 µM NA (black line) in astrocytes expressing RFP-TDP-43^wt^ (n = 55; black circles) and RFP-TDP-43^208–414^ (n = 162; gray circles). Each data point represents the mean ± SEM. (**C**,**D)** Mean peak amplitude (**C**) and cumulative Ca^2+^ response (**D**; ΔF/F_0_) upon the addition of NA for RFP-TDP-43^wt^ and RFP-TDP-43^208–414^ experimental groups. Numbers adjacent to the error bars depict the number of cells analysed. Data are presented as means ± SEM and acquired from four different animals (multiple cells were recorded per coverslip). Asterisk denotes statistical significance, *P < 0.05, determined by the Mann-Whitney U test.

Astrocytes appear to be the primary target of NA, an important neuromodulator in the CNS^28–30,40^. Through binding to ARs on the surface of astrocytes, NA activates cAMP and Ca^2+^ signaling^37,39,56^, which may be dysregulated in various neurologic disorders^32,57–60^. To study whether ALS- and FTD-U-linked TDP-43 inclusions affect cAMP and Ca^2+^ signaling, we monitored NA-induced changes in [cAMP]_i_ and [Ca^2+^]_i_ using real-time confocal microscopy and genetically encoded FRET-based cAMP nanosensor Epac1-camps^53^ or Fluo-4 AM dye, respectively, in astrocytes expressing WT (RFP-TDP-43^wt^) or mutant TDP-43 pDNA construct (RFP-TDP-43^208–414^; Figs. 3 and 4 and Online resource 1, Fig. S1).

The percentage of astrocytes co-expressing both Epac1-camps and RFP-TDP-43^wt^ or RFP-TDP-43^208–414^ construct was 68.4% (n = 54/79) or 44.7% (n = 42/94), respectively (Online resource 1, Fig. S1; Table 1). Only astrocytes that co-expressed Epac1-camps and RFP-TDP-43 pDNA constructs were used in the experiments. The addition of NA (100 µM), a non-selective α-/β-AR agonist, induced an exponential increase in the Epac1-camps FRET signal (CFP/YFP), likely reflecting a β-AR-mediated increase in [cAMP]_i_^37,39^ in both RFP-TDP-43^wt^ (n = 9) and RFP-TDP-43^208–414^-expressing astrocytes (n = 9; Fig. 3B; Table 2). The mean amplitude change in the FRET signal (mean ΔFRET [%]) and the mean time constant (τ) in astrocytes expressing RFP-TDP-43^wt^ were 27.1 ± 3.6% and 19.9 ± 2.7 s, respectively (Fig. 3C,D), not significantly different from control, Epac1-camps-expressing astrocytes (Online resource 1, Fig. S2A; *P* = 0.43 [ΔFRET] and *P* = 0.63 [τ])^37^, indicating that the overexpression of RFP-TDP-43^wt^ and RFP-tagging of the TDP-43^wt^ does not affect NA-mediated cAMP signals in astrocytes. However, the NA-mediated increase in [cAMP]_i_ was significantly reduced in RFP-TDP-43^208–414^- compared with RFP-TDP-43^wt^-expressing astrocytes, as reflected by the reduced amplitude in the FRET signal (~18% versus ~27%, respectively; *P* < 0.05; Fig. 3C). Similarly, the time constant of the FRET signal increase was significantly slower in RFP-TDP-43^208–414^- versus RFP-TDP-43^wt^-expressing astrocytes (10.7 ± 1.6 s versus 19.9 ± 2.7 s, respectively; *P* < 0.05; Fig. 3D). Resting levels of cAMP in RFP-TDP-43^208–414^- and RFP-TDP-43^wt^-expressing astrocytes were unchanged (not shown). Thus, noradrenergic stimulation leading to the β-AR-mediated increase in [cAMP]_i_ is reduced in astrocytes expressing the ALS- and FTD-U-related C-terminal fragment of TDP-43 (amino acids 208–414) compared with astrocytes expressing WT TDP-43.

**Table 1:** The percentage of astrocytes (co)expressing Epac1-camps and RFP-tagged TDP-43 constructs 30 h after transfection of cells with Epac1-camps and either RFP-TDP-43^wt^ or RFP-TDP-43^208–414^ pDNA constructs.

*n* (%)	*n* (%)	*n* (%)	*N*
**RFP-TDP-43**^**wt**^			
^a^Epac1-camps	^a^RFP-TDP-43^wt^	^b^Epac1-camps/RFP-TDP-43^wt^	All
20 (25.3)	5 (6.3)	54 (68.4)	79
**RFP-TDP-43**^**208–414**^			
^a^Epac1-camps	^a^RFP-TDP-43^208–414^	^b^Epac1-camps/RFP-TDP-43^208–414^	All
37 (39.4)	15 (16.0)	42 (44.7)	94

Epac1-camps, cAMP nanosensor; RFP-TDP-43^wt^, RFP-tagged TDP-43 wild-type; RFP-TDP-43^208–414^, RFP-tagged C-terminal fragment (amino acids 208–414) of TDP-43; *n*, number of cells.

^a^Astrocytes were expressing either Epac1-camps, RFP-TDP-43^wt^ or RFP-TDP-43^208–414^. ^b^Astrocytes were co-expressing Epac1-camps with either RFP-TDP-43^wt^ or RFP-TDP-43^208–414^.

**Table 2:** Responsiveness of astrocytes transfected with RFP-tagged TDP-43 pDNA constructs to noradrenaline- and isoprenaline-induced changes in intracellular cAMP and lactate levels.

FRET nanosensor	Experimental group	*n* (%) increase	*n* (%) decrease	*n* (%) unresponsive	*N* all
*Noradrenaline*					
Epac1-camps	RFP-TDP-43^wt^	9 (100)	0 (0)	0 (0)	9
	RFP-TDP-43^208–414^	9 (100)	0 (0)	0 (0)	9
Laconic	RFP-TDP-43^wt^	9 (42.8)	1 (4.8)	11 (52.4)	21
	RFP-TDP-43^208–414^	7 (70.0)	0 (0)	3 (30.0)	10
*Isoprenaline*					
Laconic	RFP-TDP-43^wt^	16 (64.0)	0 (0)	9 (36.0)	25
	RFP-TDP-43^208–414^	9 (60.0)	0 (0)	6 (40.0)	15

Epac1-camps, cAMP nanosensor; Laconic, lactate nanosensor; RFP-TDP-43^wt^, RFP-tagged TDP-43 wild-type pDNA construct; RFP-TDP-43^208–414^, RFP-tagged TDP-43 mutant pDNA construct; *n*, number of cells.

Next, astrocytes expressing RFP-TDP-43^wt^ or RFP-TDP-43^208–414^ were labeled with Ca^2+^ indicator, Fluo-4 AM dye, and treated with NA (100 µM; Fig. 4). Application of NA elicited rapid increase in Fluo-4 AM fluorescence (ΔF) that after reaching a peak started to decrease towards the baseline level both in RFP-TDP-43^wt^- and RFP-TDP-43^208–414^-expressing astrocytes (Fig. 4B), reflecting increase in [Ca^2+^]_i_ likely through activation of α_1_-ARs^39^. The mean peak amplitude and the cumulative Ca^2+^ response were, however, significantly lower in astrocytes expressing RFP-TDP-43^208–414^ (3.2 ± 0.3 ΔF/F_0_ [RFP-TDP-43^wt^; n = 55] versus 2.6 ± 0.2 ΔF/F_0_ [RFP-TDP-43^208–414^; n = 162; *P* < 0.05] and 98.5 ± 15.2 ΔF/F_0_ [RFP-TDP-43^wt^; n = 55] versus 68.1 ± 7.2 ΔF/F_0_ [RFP-TDP-43^208–414^; n = 162; *P* < 0.05]; respectively; Fig. 4C,D).

These results show that NA-mediated cAMP and Ca^2+^ signaling are attenuated in astrocytes with ALS- and FTD-U-linked TDP-43 inclusions.

### Responsiveness of cells to noradrenaline-induced lactate production was higher in TDP-43^208–414^- than in TDP-43^wt^-expressing astrocytes

**Figure 5:**
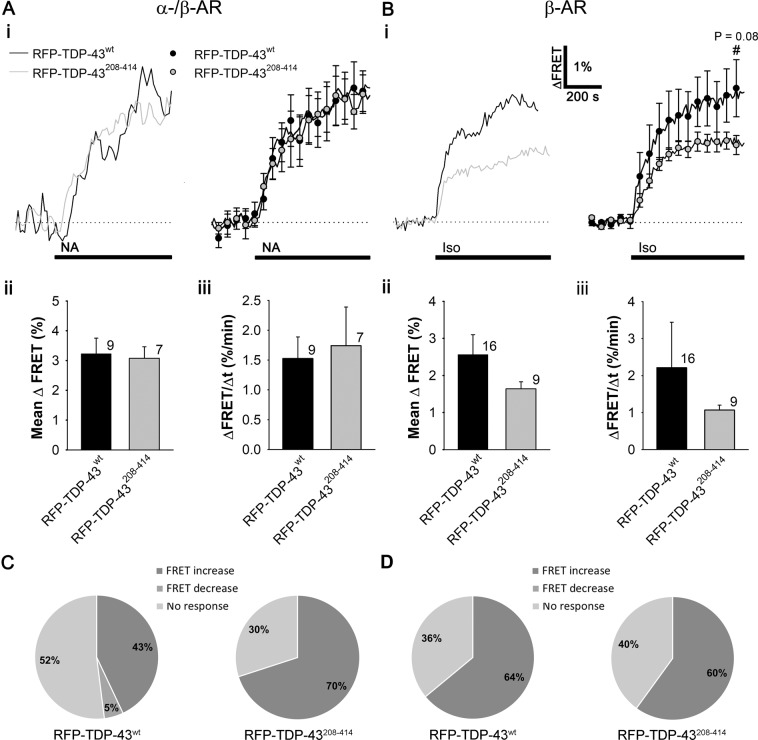
Noradrenaline- and isoprenaline-induced increase in [lactate]_i_ in astrocytes expressing RFP-TDP-43^208–414^ versus RFP-TDP-43^wt^. (**A**,**B)** panels **i** Representative (left) and average (right) time-dependent changes in the Laconic FRET signal increase (ΔFRET; mTFP/Venus) after the addition of 100 µM NA (**A**,**i**) and 100 µM Iso (**B**,**i**; black lines) in astrocytes co-expressing RFP-TDP-43^wt^ (black line/circles; n = 9 (NA), n = 16 (Iso)) or RFP-TDP-43^208–414^ (grey line/circles; n = 7 (NA), n = 9 (Iso)). Data are expressed as percentages of the inverse FRET signal (mTFP/Venus), denoting an increase in [lactate]_i_, relative to the baseline FRET signal. Each data point in the right panel represents the mean ± SEM. (**A**,**B)** panels **ii**, **iii** Mean changes in the Laconic FRET signal increase (Mean ΔFRET; (**A**,**B)** panels **ii**) and the mean initial rates of the FRET signal increase (ΔFRET/Δt; (**A**,**B)** panels **iii**) after the addition of NA (**A**) and Iso (**B**) for RFP-TDP-43^wt^ and RFP-TDP-43^208–414^ experimental groups. Numbers adjacent to the error bars depict the number of cells analysed. Data are presented as means ± SEM and acquired from at least two different animals (one cell was recorded per coverslip). The Student’s t-test, used to test significant differences between astrocytes expressing RFP-TDP-43^208–414^ and RFP-TDP-43^wt^, revealed the similarity of the responses, however, in Iso-treated astrocytes expressing RFP-TDP-43^208–414^ there was a trend towards the reduction in the [lactate]_i_ increase. (**C**,**D)** Pie graphs showing the responsiveness of astrocytes to (**C**) noradrenaline- and (**D**) isoprenaline-induced changes in intracellular lactate levels. Note that the probability of observing a response to NA with production of lactate in RFP-TDP-43^208–414^-expressing astrocytes was 1.6-fold higher compared to RFP-TDP-43^wt^-expressing astrocytes, but not in Iso-treated cells (see also Table 2).

In astrocytes, noradrenergic signaling regulates glucose metabolism, in particular aerobic glycolysis and lactate production^40,61,62^, which may be altered in ALS^52^. This may affect astrocyte-neuron lactate shuttle and astrocytic metabolic support of neurons. Because NA-mediated cAMP signaling is reduced in astrocytes overexpressing RFP-TDP-43^208–414^, we asked whether RFP-TDP-43^208–414^ overexpression affects the NA-mediated increase in aerobic glycolysis and therefore lactate production. To monitor real-time changes in [lactate]_i_, upon the addition of NA, cultured rat astrocytes expressing RFP-TDP-43^wt^ or RFP-TDP-43^208–414^ were co-transfected with the FRET-based nanosensor Laconic (Fig. 5)^54^. As in the experiments with Epac1-camps, only astrocytes expressing both Laconic and RFP-TDP-43 pDNA constructs were used in the analysis.

The results revealed that the amplitudes (ΔFRET [%]) and the initial rates of the Laconic FRET signal increase (ΔFRET/Δtime [%/min]) in NA-responsive cells were similar in the RFP-TDP-43^wt^- and the RFP-TDP-43^208–414^-expressing astrocytes (3.2 ± 0.5% [RFP-TDP-43^wt^; n = 9] versus 3.1 ± 0.4% [RFP-TDP-43^208–414^; n = 7; *P* = 0.83] and 1.5 ± 0.4%/min [RFP-TDP-43^wt^; n = 9] versus 1.7 ± 0.6%/min [RFP-TDP-43^208–414^; n = 7; *P* = 0.77]; respectively; Fig. 5A, Table 2). In control experiments, in which isolated astrocytes were transfected with Laconic only (no co-transfection with RFP-TDP-43 constructs), stimulation with NA (100 µM) elicited similar increases in [lactate]_i_ as in astrocytes expressing RFP-TDP-43^wt^ (Online resource 1, Fig. S2B; n = 7 cells), indicating that overexpression of RFP-TDP-43^wt^ does not affect the normal glycolytic response in astrocytes.

However, when we looked at the whole population of astrocytes, we observed that the responsiveness of astrocytes to NA-induced lactate production increased by 1.6-fold (from 42.8% to 70.0%) in cells expressing RFP-TDP-43^208–414^ compared with astrocytes expressing RFP-TDP-43^wt^ (Fig. 5C; Table 2), suggesting that the probability of activating aerobic glycolysis in a cell population is facilitated in astrocytes with TDP-43 cytoplasmic inclusions. In control astrocytes expressing only Laconic (no co-transfection with RFP-TDP-43 constructs), the responsiveness of astrocytes to NA-induced lactate production was similar to that in astrocytes expressing RFP-TDP-43^wt^ (31.8% versus 42.8%, respectively).

When we treated astrocytes with the selective β-AR agonist Iso, the responsiveness of cells was similar (64.0% (RFP-TDP-43^wt^) versus 60.0% (RFP-TDP-43^208–414^); Table 2, Fig. 5D). The mean amplitude and the initial rate of the Laconic FRET signal increase were 1.6-fold and 2-fold lower, respectively, but statistically insignificantly different, in the RFP-TDP-43^208–414^ than in the RFP-TDP-43^wt^-expressing astrocytes (2.6 ± 0.5% [RFP-TDP-43^wt^; n = 16] versus 1.6 ± 0.2% [RFP-TDP-43^208–414^; n = 9; *P* = 0.2] and 2.2 ± 1.2%/min [RFP-TDP-43^wt^; n = 16] versus 1.1 ± 0.1%/min [RFP-TDP-43^208–414^; n = 9; *P* = 0.4]; respectively; Fig. 5B, Table 2).

### The overexpression of TDP-43^208–414^ reduces the expression of β_2_-adrenergic receptors in astrocytes

**Figure 6:**
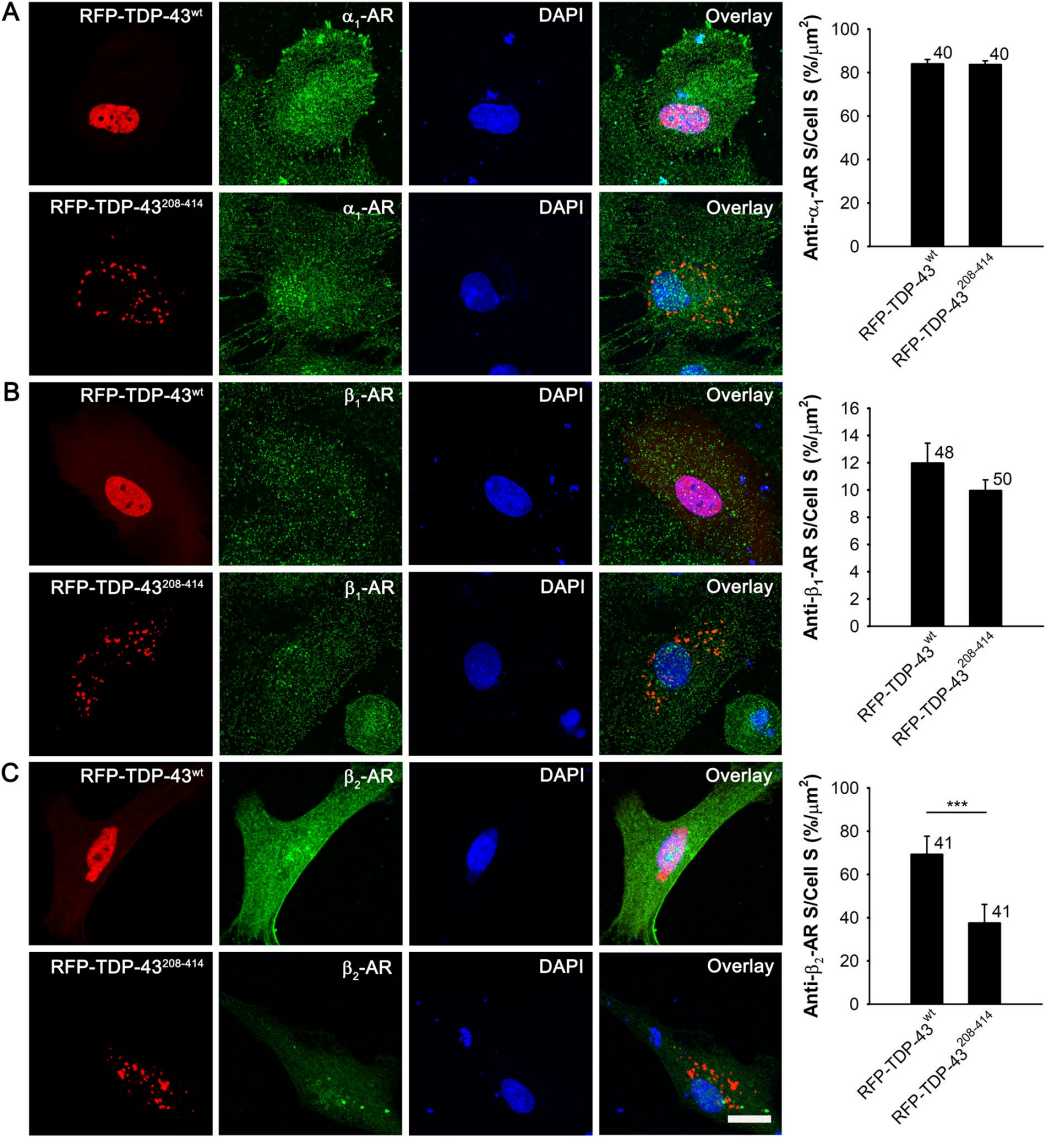
Reduced expression of β_2_-adrenergic receptors in RFP-TDP-43^208–414^- compared with RFP-TDP-43^wt^-expressing astrocytes. (**A**–**C)** Representative fluorescence images of astrocytes immunostained with α_1_- (**A**) β_1_- (**B**) and β_2_-adrenergic receptor (**C**) antibodies (green) and labelled with DAPI (blue) in RFP-TDP-43^wt^- (upper panels) and RFP-TDP-43^208–414^-expressing astrocytes (lower panels; red). Immunostaining was performed 30 h after transfection with RFP-tagged TDP-43 pDNA constructs. Scale bar, 20 µm. Histograms show anti-α_1_- (**A**) anti-β_1_- (**B**) and anti-β_2_-adrenergic receptor (AR; **C**) positive cell cross-sectional area (anti-α_1_-*/*β_1_-*/*β_2_-AR S; i.e. number of green fluorescence pixels with fluorescence intensity above the threshold of 10% of maximal fluorescence) per total cell cross-sectional area (cell S; i.e. number of all pixels) in RFP-TDP-43^wt^- and RFP-TDP-43^208–414^-expressing cells. Note the reduced expression of β_2_-adrenergic receptors in RFP-TDP-43^208–414^- compared with RFP-TDP-43^wt^-expressing astrocytes. Numbers adjacent to the error bars depict the number of cells analysed. Data are presented as means ± SEM and acquired from at least two different animals. Each experiment (*i*.*e*. coverslip) was performed in duplicate, multiple cells were recorded per coverslip. ***P ≤ 0.001, Mann-Whitney U test.

Because the expression of RFP-TDP-43^208–414^ causes a reduction in NA-mediated cAMP and Ca^2+^ signaling compared with cells expressing RFP-TDP-43^wt^, we next investigated whether the overexpression of RFP-TDP-43^208–414^ alters the expression of ARs. Astrocytes express all types of ARs^33,34,63^. We immunostained astrocytes expressing RFP-TDP-43^wt^ and RFP-TDP-43^208–414^ with antibodies against α_1_-, β_1_-, or β_2_-ARs (Fig. 6).

While the expression of α_1_- and β_1_-ARs was not significantly different between the RFP-TDP-43^wt^- and RFP-TDP-43^208–414^-expressing astrocytes (84.0% ± 2.0% [n = 40] versus 83.7% ± 1.6% [n = 40; *P* = 0.63]) and 12.0% ± 1.4% [n = 48] versus 10.0% ± 0.8% [n = 50; *P* = 0.62], respectively; Fig. 6A,B), the expression of β_2_-ARs was significantly lower in astrocytes expressing RFP-TDP-43^208–414^ (69.2% ± 8.4% [RFP-TDP-43^wt^; n = 41] versus 37.5% ± 8.6% [RFP-TDP-43^208–414^; n = 41]; *P* < 0.001; Fig. 6C). This indicates a downregulation of β_2_-ARs in astrocytes expressing the ALS- and FTD-U-linked RFP-TDP-43^208–414^ construct, which may contribute to the reduced intracellular cAMP and Ca^2+^ signaling (Figs. 3 and 4) in these cells^39^.

### The expression of MCT1 transporters is reduced in TDP-43^208–414^-expressing astrocytes

**Figure 7:**
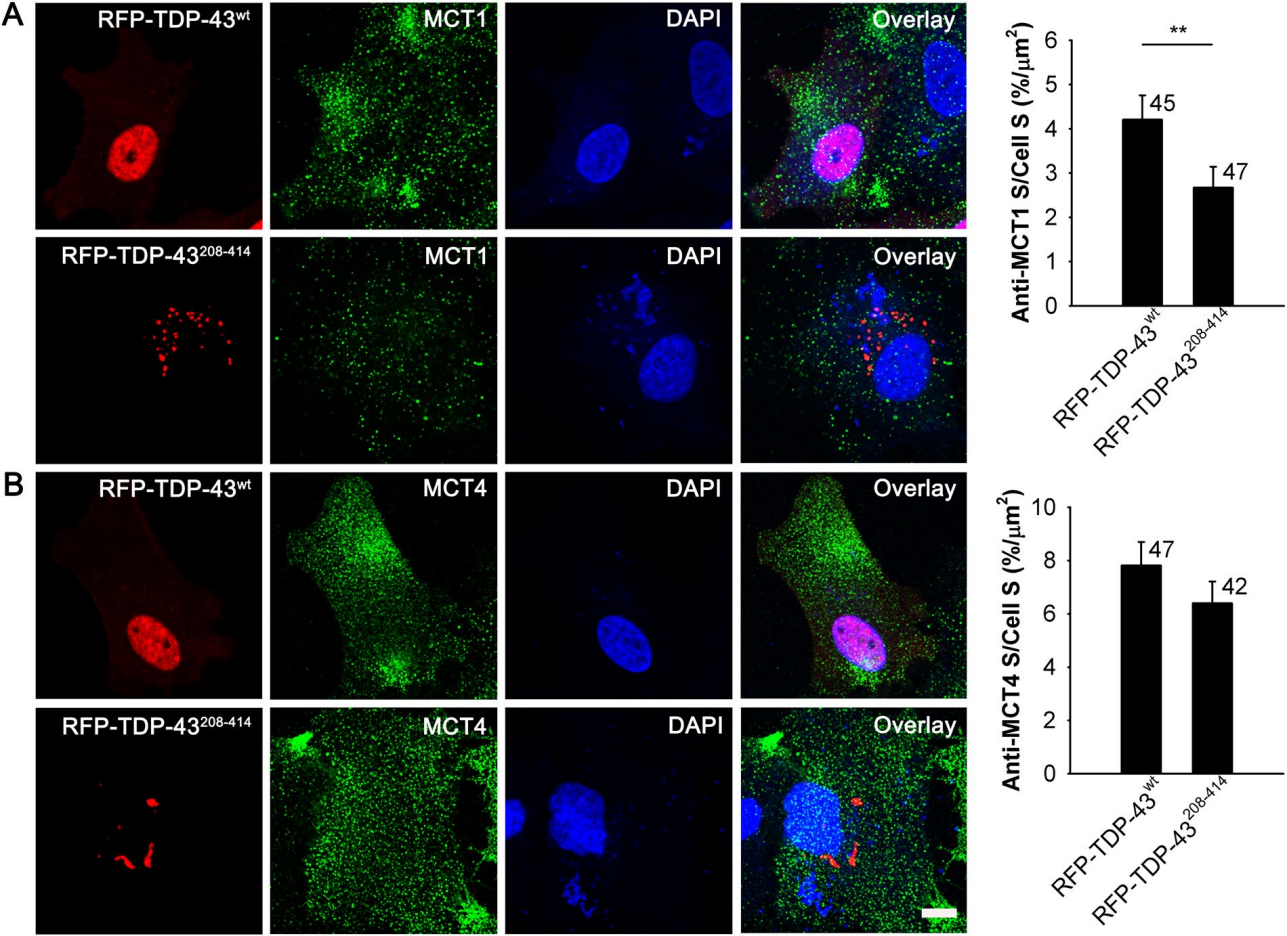
Reduced expression of MCT1 transporters in RFP-TDP-43^208–414^- compared with RFP-TDP-43^wt^-expressing astrocytes. (**A**,**B**) Representative fluorescence images of astrocytes immunostained with antibodies against MCT1 (**A**) and MCT4 transporters (**B**, green) and labelled with DAPI (blue) in RFP-TDP-43^wt^- (upper panels) and RFP-TDP-43^208–414^-expressing astrocytes (lower panels; red). Immunostaining was performed 24 h after transfection with RFP-tagged TDP-43 pDNA constructs. Scale bar, 10 µm. Histograms show anti-MCT1 (**A**) and anti-MCT4 (**B**) positive cell cross-sectional area (anti-MCT1*/*MCT4 S; i.e. number of green fluorescence pixels with fluorescence intensity above the threshold of 20% of maximal fluorescence) per total cell cross-sectional area (cell S; i.e. number of all pixels) in RFP-TDP-43^wt^- and RFP-TDP-43^208–414^-expressing cells. Note the reduced expression of MCT1 transporters in RFP-TDP-43^208–414^- compared with RFP-TDP-43^wt^-expressing astrocytes. Numbers adjacent to the error bars depict the number of cells analysed. Data are presented as means ± SEM and acquired from four different animals. Each experiment (*i*.*e*. coverslip) was performed in duplicate, multiple cells were recorded per coverslip. **P ≤ 0.01, Mann-Whitney U test.

Since NA-mediated lactate production was enhanced in astrocytes with TDP-43 inclusions (Fig. 5), which may affect the availability of extracellular lactate, we next investigated whether the presence of TDP-43 inclusions alters the expression level of astrocyte-specific monocarboxylate transporters (MCT), which are in astrocytes predominantly responsible for L-lactate transport across the plasma membrane. We immunostained astrocytes expressing RFP-TDP-43^wt^ and RFP-TDP-43^208–414^ with antibodies against MCT1 and MCT4 transporters (Fig. 7). The expression level of MCT4 transporters did not significantly differ between the RFT-TDP-43^wt^- and RFP-TDP-43^208–414^-expressing astrocytes (7.8% ± 0.9% [n = 47] versus 6.4% ± 0.8% [n = 42; *P* = 0.25]; Fig. 7B), while the expression of MCT1 transporters was significantly lower in astrocytes expressing RFP-TDP-43^208–414^ (4.2% ± 0.5% [n = 45] versus 2.7% ± 0.5% [n = 47; *P* < 0.01]; Fig. 7A). These results suggest a downregulation of MCT1 transporters in astrocytes with ALS- and FTD-U-linked TDP-43 inclusions, which indicates a decreased astroglial lactate release capacity.

## Discussion

Based on the new disease models, a number of recent studies have highlighted the involvement of non-neuronal cells in the pathogenesis of ALS and FTD-U, including astrocytes^2,7^, which provide metabolic and trophic support to motor neurons. However, the molecular mechanisms of astroglial-mediated neurotoxicity in ALS and FTD-U remain poorly understood. Here, we investigated whether astroglial expression of the C-terminal fragment of TDP-43 (TDP-43^208–414^), a major component of ALS- and FTD-U-associated pathologic cytoplasmic inclusions, affects astroglial cell metabolism. TDP-43 inclusions may compromise astroglial metabolic support of neurons in ALS and FTD-U and contribute to CNS hypometabolism observed in patients with neurodegeneration.

Expression of C-terminal fragment of TDP-43 in isolated cortical rat astrocytes has led to the formation of cytoplasmic TDP-43 inclusions, which can mimic key biochemical features of TDP-43 proteinopathies^10,20^, consistent with studies in other cell types^20,64^. Astrocytes with cytoplasmic TDP-43 inclusions had a 3-fold lower amount of endogenous nuclear TDP-43 (which may cause a partial loss-of-function of nuclear TDP-43) compared with astrocytes expressing WT TDP-43, where most of the TDP-43 was present in the cell nucleus. This indicates that expression of the C-terminal fragment of TDP-43 in astrocytes affects trafficking of endogenous TDP-43 between the nucleus and the cytoplasm, consistent with reports on neurons^5^, glial cells from human tissue samples^55^, various cell lines (e.g. QBI-293 and tsBN2 cells^10^), and muscle cells^65^. The results suggest that aggregated TDP-43 prevents newly synthesized endogenous TDP-43 from being imported into the nucleus and/or inhibits the re-entry of existing cytoplasmic TDP-43 into the nucleus^10,13^. Interestingly, the transfection with inclusion-forming TDP-43, caused in neighboring non-transfected astrocytes (with no visible RFP signal) a small, albeit not significant, trend in reduced percentage of DAPI and TDP-43-colabeled nuclei, which suggests that the transfection with the C-terminal fragment of TDP-43 might affect the physiology of non-transfected cells. In pathological conditions, mimicked here by the expression of inclusion-forming TDP-43, astrocytes bear a significant functional plasticity, known as reactive astrogliosis^66^. These astrocytes secrete or distribute through gap junctions various cytotoxic factors, such as Lcn2^22,67,68^, interleukin-6, ciliary neurotrophic factor, etc.^69^, which can affect neighboring cells. Even though we did not specifically test whether astrocytes expressing inclusion-forming TDP-43 transform into a reactive phenotype, this outcome is possible. If this is the case, the observed reduction in the nuclear TDP-43 staining in some non-transfected astrocytes adjacent to inclusion-forming TDP-43 astrocytes may be a consequence of altered physiological state of these cells affecting the TDP-43 gene expression level or the synthesis/degradation/distribution of TDP-43.

Because TDP-43 is involved in multiple aspects of RNA processing, any changes in TDP-43 nuclear level may have detrimental effects on astroglial physiology^3^, including on cell metabolism. LD accumulation has been observed in glial cells, including astrocytes, in early stages of neurodegeneration^46^. Recently, alterations in lipid metabolism (accumulation of cholesteryl esters, determined with lipidomic analysis) in spinal cords from SOD1^G93A^ transgenic mouse model have been reported that might be linked to astrogliosis and LD formation in astrocytes^48^, since a population of astrocytes isolated from the degenerating spinal cords of the same animal model exhibited significant abundance of LDs as well as autophagic and secretory vesicles, all characteristic features of cellular stress and inflammatory activation^70^. However, the mechanisms leading to increased LD accumulation in astrocytes are poorly understood and may, among others, rely on altered neuronal mitochondrial function and ROS as well as on astrocyte-neuron lactate shuttle^47^, in particular on astroglial lactate-derived lipid production in neurons and transfer of excess lipids in lipoprotein-like particles (ApoE) from neurons to astrocytes^47,71^. We show here that in astrocytes with TDP-43 inclusions the LD presence is enhanced (the size and the number of LDs) in the absence of neighboring neurons, suggesting the existence of an alternative astroglial-mediated mechanism triggering accumulation of LDs through altering the balance between biogenesis and degradation of LDs. Accumulation of LDs in astrocytes with cytoplasmic TDP-43 inclusions may be a response to cellular inflammation, which is typically found in the pathology of various neurologic disorders, including ALS^66,72^. Here, LDs are hypothesized to be an important source of energy for proliferation and may serve a protective role by gathering free fatty acids to protect cells against lipotoxicity^73^.

Besides alterations in LD metabolism, changes in noradrenergic regulation of glucose metabolism were observed in astrocytes with cytoplasmic TDP-43 inclusions. Astrocytes with TDP-43 inclusions exhibited downregulation of β_2_-ARs and a 35% reduction in NA-mediated increase in [cAMP]_i_. Consistent with our results, dysregulation of astrocytic β_2_-AR/cAMP signalling has been suspected to contribute to the pathology of a number of other neurologic disorders, including multiple sclerosis, Alzheimer’s disease, human immunodeficiency virus encephalitis, and others^74^. Reduction of β_2_-AR expression was reported in human white matter astrocytes obtained from post mortem brain tissue of patients with multiple sclerosis^57,75^. Moreover, it was demonstrated both *in vitro* and *in vivo* that the presence of Alzheimer’s disease associated amyloid beta peptide (Aβ) in prefrontal cortical neurons leads to internalization and degradation of β_2_-ARs, which leads to subsequent attenuation of cAMP signalling^59^. Since aerobic glycolysis in astrocytes is upregulated with β-AR/cAMP signaling, one would expect that astrocytes with TDP-43 inclusions and a reduced expression of β_2_-ARs will exhibit reduced β-adrenergic mediated aerobic glycolysis and lactate production. When we stimulated astrocytes with Iso, a selective β-AR agonist, although the responsiveness of cells to Iso was unchanged, there was a trend in reduction of [lactate]_i_ increase, since the amplitude in [lactate]_i_ increase was ~2-fold lower (*P* = 0.08) in astrocytes with TDP-43 inclusions, consistent with downregulation of β_2_-AR and cAMP signaling in these cells. In contrast to Iso stimulation, the amplitude and the rate of [lactate]_i_ increase upon NA stimulation was unaltered in astrocytes with TDP-43 inclusions, but the probability of activating aerobic glycolysis, measured as increased responsiveness to NA, was increased by 1.6-fold in astrocytes with TDP-43 inclusions. When viewing the astroglial population as a whole, this means that glycolytic lactate production upon NA stimulation is facilitated in astrocytes with TDP-43 inclusions.

Besides cAMP, Ca^2+^ signals through activation of α_1_-AR/G_q_-protein signaling pathway have important role in regulation of NA-mediated glucose metabolism in astrocytes^33^. Abnormal Ca^2+^ homeostasis has been observed in astrocytes isolated from SOD1^G93A^ animals. In particular, excess Ca^2+^ release from ER stores upon purinergic/G_q_-protein signaling pathway activation has been reported due to abnormal ER Ca^2+^ accumulation^76^. If such a mechanism exists in astrocytes with TDP-43 inclusions, binding of NA to α_1_-AR may lead to excess Ca^2+^-release from ER and enhanced aerobic glycolysis despite downregulation of β_2_-AR/cAMP signaling pathway. However, contrary to the results obtained on SOD1^G93A^ animal model, we observed a 31% reduction in Ca^2+^ signaling in astrocytes with TDP-43 inclusions, even though the level of α_1_-AR expression was unchanged. It has been reported that β-AR/cAMP and α_1_-AR/Ca^2+^ signaling pathways interact in astrocytes, enhancing each other^39^, therefore downregulation of β_2_-ARs may reduce the noradrenergic Ca^2+^ response via α_1_-ARs, explaining the reduction of both noradrenergic cAMP and Ca^2+^ signals despite an unaltered expression of α_1_-ARs.

It has been reported that morphologic changes in astrocytes exhibit a bell-shaped dependency on [cAMP]_i_^37^. If aerobic glycolysis in astrocytes displays a similar bell-shaped dependency on [cAMP]_i_, astrocytes with reduced β_2_-AR/cAMP signalling may actually attain enhanced metabolic responsiveness to noradrenergic stimulation. Moreover, perturbances in TDP-43 may change the transcriptome and proteome of a cell affecting the expression level of enzymes involved in LD metabolism and aerobic glycolysis, as was observed in other cell types with a TDP-43 knock down^16^. Whether expression level of metabolic enzymes is altered in astrocytes with TDP-43 inclusions and whether this contributes to the observed enhanced astroglial metabolism needs to be investigated in the future.

Increased lactate production upon NA stimulation in astrocytes with TDP-43 inclusions may lead to a better metabolic support of neurons with lactate due to lactate flux generated between astrocytes and neurons^77^. This is, however, contradictory to the CNS hypometabolism observed in patients with neurodegenerative diseases, including ALS^43,45^. Recently, decreased expression of lactate MCT1 and MCT4 transporters has been reported post mortem in the motor cortex of ALS patients compared to non-ALS patients^78^. Moreover, downregulation of MCT1 mRNA in the spinal cords of early symptomatic and endstage SOD1^G93A^ transgenic mice model of ALS has been observed, presumably in glia (oligodendrocytes and astrocytes)^78^. Down-regulation of the lactate MCT4 transporter has been also demonstrated in spinal cord astrocytes from patients with ALS with SOD1 mutations and in pre-symptomatic SOD1^G93A^ transgenic mice^43^. We show here that expression of C-terminal fragment of TDP-43 (TDP-43^208–414^) *per se* in astrocytes causes a reduction in the expression of astroglial lactate MCT1 transporters. Lower expression of astroglial MCTs may decrease the lactate release capacity in astrocytes. Thus, despite facilitated NA-mediated aerobic glycolysis in astrocytes with TDP-43 inclusions, lactate may accumulate inside cells, and the metabolic support of neurons in patients may be decreased significantly contributing to neuron degeneration in ALS and FTD-U. Consistent with this hypothesis, cAMP signaling and lactate homeostasis in the brain were noted to be reduced in patients with some forms of ALS^43,45^.

**Figure 8:**
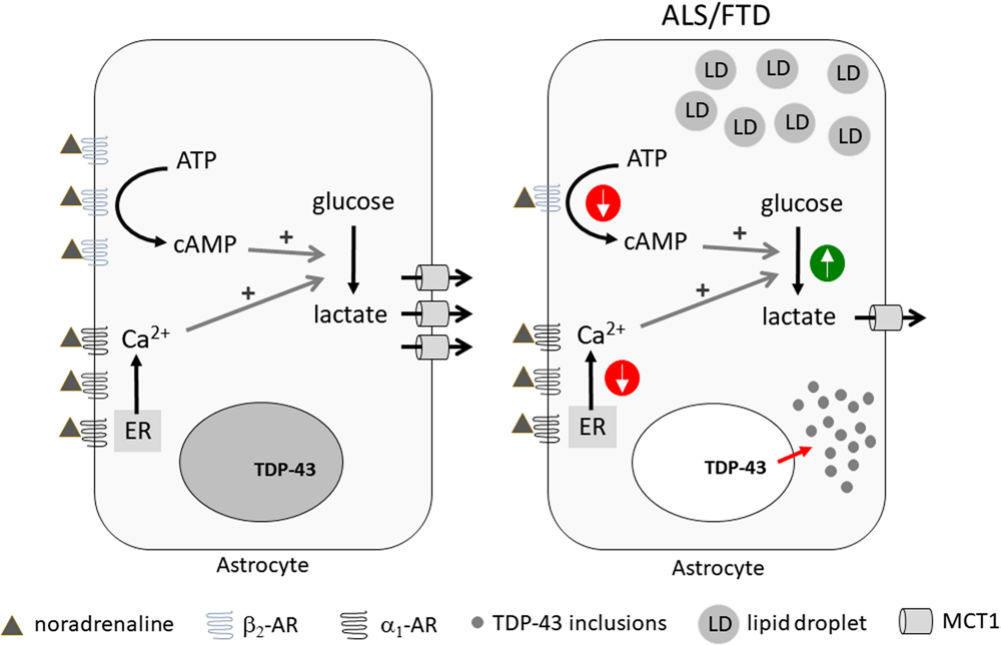
Astrocytes with TDP-43 inclusions have reduced noradrenergic signaling and dysregulated astroglial metabolism. Noradrenergic cAMP and Ca^2+^ signaling is downregulated in astrocytes expressing the ALS- and FTD-U-associated inclusion-forming C-terminal fragment of TDP-43 (TDP-43^208–414^) compared to astrocytes expressing TDP-43^wt^ in the nucleus, possibly due to downregulation of β_2_-adrenergic receptor (β_2_-AR). In these cells aerobic glycolysis and lipid droplet (LD) accumulation are facilitated representing cellular stress. Moreover, monocarboxylate transporter 1 (MCT1) is downregulated in astrocytes with TDP-43 inclusions, suggesting that despite increased astroglial aerobic glycolysis astroglial L-lactate support of neurons may be reduced in ALS or FTD-U. ALS, amyotrophic lateral sclerosis; ER, endoplasmic reticulum; FTD, frontotemporal dementia with ubiquitin-positive inclusions (FTD-U); TDP-43, TAR DNA-binding protein 43.

In conclusion, the results of the present study show that expression of the C-terminal fragment of TDP-43 in isolated rat cortical astrocytes leads to the formation of TDP-43-positive cytoplasmic inclusions, a hallmark of ALS. These astrocytes exhibit reduced NA-mediated cAMP and Ca^2+^ signaling, whereas both aerobic glycolysis and LD presence appear facilitated. Although NA-mediated L-lactate production was increased in astrocytes with TDP-43 inclusions, the expression of lactate MCT1 transporters was reduced, suggesting decreased astroglial lactate release capacity (Fig. 8). Thus, these findings reflect an astroglial stressed state that may fail to adequately metabolically support neurons in ALS and FTD-U, leading to neurotoxicity.

## Methods

### Cell culture and transfection

Unless noted otherwise, all chemicals were of the highest quality available and purchased from Sigma-Aldrich (Merck KGaA, Darmstadt, Germany).

Primary astrocyte cultures were prepared from the cerebral cortices of 2- to 3-day-old Wistar rats, as described previously^79^, and grown in high-glucose Dulbecco’s modified Eagle’s medium supplemented with 10% fetal bovine serum, 1 mM sodium pyruvate, 2 mM l-glutamine and 25 µg/ml penicillin-streptomycin in a vaporized atmosphere containing 95% air and 5% CO_2_ at 37 °C until reaching 70%–80% confluence. Confluent cultures were shaken overnight at 225 rpm and the medium was changed the next morning; this was repeated three times. After the third overnight shaking, the cells were trypsinized and put in flat tissue culture tubes with 10 cm^2^ growth area. After reaching confluence again, the cells were subcultured onto 22-mm diameter poly-l-lysine-coated glass coverslips. This procedure yielded astrocytes with > 95% purity, determined by anti-glial fibrillary acidic protein (GFAP) antibody staining^80^.

After 1–3 days, astrocytes were co-transfected with the genetically encoded FRET-based cAMP nanosensor Epac1-camps^53^ or lactate nanosensor Laconic^54^ and RFP-tagged WT TDP-43 (pTagRFP-C::TDP-43^wt^ [RFP-TDP-43^wt^]) or RFP-tagged C-terminal fragment of TDP-43 (pTagRFP-C::TDP-43^208–414^ [RFP-TDP-43^208–414^]) using FuGENE 6 Transfection Reagent (Promega Corporation, Madison, WI, USA). Transfection medium contained no serum or antibiotics.

The experimental animals were cared for in accordance with the International Guiding Principles for Biomedical Research Involving Animals developed by the Council for International Organizations of Medical Sciences and the Animal Protection Act (Official Gazette RS, no. 38/13). The experimental protocol was approved by The Administration of the Republic of Slovenia for Food Safety, Veterinary and Plant Protection (Republic of Slovenia, Ministry of Agriculture, Forestry and Food, Dunajska cesta 22, 1000 Ljubljana), Document No. U34401-47/2014/7, signed by Barbara Tomše, DVM. Every set of data was acquired from at least 2 independent experiments.

### Immunocytochemistry and data analysis

Control (non-transfected) astrocytes and astrocytes expressing RFP-TDP-43^wt^ or RFP-TDP-43^208–414^ were fixed with 4% formaldehyde in phosphate buffered saline (PBS) for 15 min and then permeabilized with 0.1% Triton X-100 for 10 min at room temperature (except for MCT1 and MCT4) before being treated with 10% goat serum for 1 h at 37 °C. Astrocytes were then stained with primary antibodies against endogenous TDP-43 (C-terminal, rabbit polyclonal, 1:400; Proteintech, Manchester, UK), α_1_-AR (rabbit polyclonal, 1:100; Abcam, Cambridge, UK), β_1_-AR (rabbit polyclonal, 1:200; Abcam, Cambridge, UK), β_2_-AR (rabbit polyclonal, 1:100; Biorbyt, Cambridge, UK), MCT1 (rabbit polyclonal, 1:50; Abcam, Cambridge, UK) and MCT4 (rabbit polyclonal, 1:50; Santa Cruz, Texas, US) overnight at 4 °C. After washing with PBS, cells were incubated for 1 h at 37 °C with Alexa Fluor^488^-conjugated secondary goat anti-rabbit IgG (1:600; Abcam, Cambridge, UK). Excess antibodies were washed off and the coverslips were mounted onto glass slides using SlowFade antifade reagent with DAPI (Molecular Probes by Life Technologies, Thermo Fisher Scientific, Massachusetts, USA) and carefully sealed. Immunolabelled cells were imaged with an inverted Zeiss LSM780 confocal microscope with a Plan apochromatic 40×/1.4 oil immersion objective (Carl Zeiss, Jena, Germany) using 488-nm Ar-ion, 543-nm He-Ne and 405-nm diode laser excitation. Emission spectra were acquired sequentially with 505- to 530-nm bandpass (Alexa Fluor^488^), 560-nm long pass (TagRFP^584^) and 445- to 450-nm bandpass (DAPI) emission filters.

Colocalization analysis of Alexa Fluor^488^ (immunolabelled TDP-43), TagRFP^584^ (RFP-TDP-43 pDNA constructs), and DAPI fluorescence signals was performed on exported TIFF files using ColocAna software (Celica Biomedical, Ljubljana, Slovenia)^81^ that counts all red, green, blue and colocalized (green and red or green and blue) pixels within the image. The threshold for the colocalized pixel count was set at 20% of the maximal green, red or blue fluorescence intensity, respectively. Colocalization was expressed as the ratio of colocalized to green or blue pixels (as percentages). Kruskal-Wallis one-way analysis of variance (ANOVA) on ranks (followed by Dunn’s test) or Mann-Whitney U test were performed to determine statistical significance between the experimental groups. *P* < 0.05 was considered significant.

To determine the expression level of different ARs and MCTs, the number of α_1_-AR-, β_1_-AR-, β_2_-AR-, MCT1- and MCT4-positive green fluorescence pixels with fluorescence intensity above the threshold of 10% (α_1_-/β_1_-/β_2_-AR-positive cell area) or 20% (MCT1 and MCT4-positive cell area) of maximal fluorescence and the number of all pixels (total cell area) per cell cross-sectional area were determined for each cell separately in RFP-TDP-43^wt^- and RFP-TDP-43^208–414^-expressing astrocytes using ZEN software (Zeiss, Oberkochen, Germany). The percentage of the AR- and MCT-positive cell area relative to the total cell area was calculated for RFP-TDP-43^wt^- and RFP-TDP-43^208–414^-expressing astrocytes for individual receptor and transporter type. The Mann-Whitney U test was performed to determine statistical significance between the experimental groups. *P* < 0.05 was considered significant.

To determine the extent of DAPI and TDP-43-colabeled nuclei in non-transfected astrocytes (with no visible RFP signal) adjacent to the RFP-TDP-43^wt^- and RFP-TDP-43^208–414^-expressing astrocytes (with visible RFP signal) we measured the percentage of DAPI and TDP-43-colabeled nuclei per all DAPI nuclei in neighboring non-transfected cells (with no visible RFP signal) in the RFP-TDP-43^wt^ and RFP-TDP-43^208–414^ experimental groups. In the control non-transfected group, we analyzed the nuclei of all cells. Some partially visible DAPI-stained nuclei at the edges of the confocal images were also taken into account in the analysis, however, only if no RFP signal in or around the visible part of the nuclei was observed. Kruskal-Wallis one-way ANOVA on ranks, followed by Dunn’s test was performed to determine statistical significance between the experimental groups. *P* < 0.05 was considered significant.

### Lipid droplet staining and data analysis

Control (non-transfected) astrocytes and astrocytes transfected with RFP-TDP-43^wt^ or RFP-TDP-43^208–414^ plasmid were incubated in fresh growth medium for 24 h. Cells were then fixed in 4% formaldehyde in PBS for 5 min and stained with 1 µg/ml BODIPY^493/503^ (Molecular Probes by Life Technologies, Thermo Fisher Scientific, Massachusetts, USA), a fluorescent LD marker, for 6 min at room temperature. Excess dye was washed off and the coverslips were mounted onto glass slides using SlowFade antifade reagent with DAPI (Molecular Probes by Life Technologies, Thermo Fisher Scientific, Massachusetts, USA) and carefully sealed. Stained cells were imaged with an inverted Zeiss LSM780 confocal microscope with a Plan apochromatic 40×/1.4 oil immersion objective (Carl Zeiss, Jena, Germany) using 488-nm Ar-ion, 543-nm He-Ne and 405-nm diode laser excitation. Emission spectra were acquired sequentially with 505- to 530-nm bandpass (BODIPY^493/503^), 560-nm long pass (TagRFP^584^) and 445- to 450-nm bandpass (DAPI) emission filters.

To determine the LD content in individual astrocytes, the number of BODIPY^493/503^-positive green fluorescence pixels with fluorescence intensity above the threshold of 20% of maximal fluorescence (Lipid droplet S) and the number of all pixels (Cell S) were determined for each cell separately in control, RFP-TDP-43^wt^- and RFP-TDP-43^208–414^-expressing astrocytes in ZEN software (Zeiss, Oberkochen, Germany). The values obtained were normalized to the average LD content in control non-transfected cells. The mean number of BODIPY^493/503^-labeled LDs per cell and the mean LD perimeter were determined in cross-sections of individual cells using ImageJ, Analyze Particles function after applying 20% threshold and signal intensity (watershed) segmentation on individual images. Then, assuming that all LDs are spherical, the mean LD diameter was estimated from the perimeter values with the equation *d* = *C*/π, where *d* is the diameter and *C* is the LD perimeter. Kruskal-Wallis one-way ANOVA on ranks, followed by Dunn’s test was performed to determine statistical significance between the groups. *P* < 0.05 was considered significant.

### FRET measurements of intracellular cAMP and lactate levels and data analysis

Cells co-expressing the FRET-based nanosensor Epac1-camps or Laconic and RFP-tagged TDP-43 pDNA construct (RFP-TDP-43^wt^ or RFP-TDP-43^208–414^) were examined 24–30 h after transfection with a fluorescence microscope Zeiss Axio Obsever.A1 (Zeiss, Oberkochen, Germany), with a CCD camera and monochromator Polychrome V (Till Photonics, Graefelfing, Germany) as a monochromatic source of light with a wavelength 436 nm/10 nm. Dual emission intensity ratios were recorded using an image splitter (Optical Insights, Tucson, AZ, USA) and two emission filters (465/30 nm for CFP [cyan fluorescent protein] or mTFP [monomeric teal fluorescent protein] and 535/30 nm for YFP [yellow fluorescent protein] or Venus). Images were acquired every 3.5 s for Epac1-camps and 10 s for Laconic. Exposure time was 100 ms.

In some experiments astrocytes co-expressing Laconic and RFP-TDP-43^wt^ or RFP-TDP-43^208–414^ were examined 24 h after transfection with a fluorescence microscope (Zeiss Axio Observer.A1 (Zeiss, Oberkochen, Germany)) with a Axiocam 702 camera and Colibri.2 Lamp Module (Zeiss, Oberkochen, Germany) as a source of light with a wavelength 433 nm. Dual emission intensity ratios were recorded using an image splitter (Optical Insights, Tucson, AZ, USA) and two emission filters (469-491 nm for ECFP and 530-4095 nm for EYFP). Images were acquired at intervals of 10 s with exposure time of 100 ms.

Coverslips with transfected astrocytes were mounted in a superfusion recording chamber on the microscope stage. Imaging was performed at room temperature (22–24 °C). One cell per experiment was recorded. The FRET signals (CFP/YFP (Epac1-camps) and mTFP/Venus (Laconic) signals) were obtained from the integration of the ratio signal over the entire cell using Life Acquisition software (Till Photonics, Graefelfing, Germany) or ZEN (Carl Zeiss, Jena, Germany). In the graphs, the FRET signal was reported as the ratio of the CFP/YFP (Epac1-camps) and mTFP/Venus (Laconic) fluorescence signals after subtracting the background fluorescence from the individual fluorescence signals using Excel (Microsoft, Seattle, WA, USA). The values of the FRET ratio signals were normalized to 1.0. An increase in the FRET ratio signal reflects an increase in [cAMP]_i_ or [lactate]_i_.

Before the experiments, astrocytes were kept in extracellular solution containing sodium bicarbonate for 10 min (10 mM Hepes/NaOH [pH 7.2], 3 mM d-glucose, 135.3 mM NaCl, 1.8 mM CaCl_2_, 2 mM MgCl_2_, and 5 mM KCl, 0.5 mM NaH_2_PO_4_·H_2_O, 5 mM NaHCO_3_), and then treated with 100 µM NA (non-selective AR agonist) or 100 µM Iso (selective β-AR agonist) following a 100- to 200-s baseline. Experiments were conducted with the addition of a bolus solution; 200 µl of extracellular solution containing NA was added by pipette to 200 µl of bath solution in the recording chamber. The application of a control bolus solution without reagents had no significant effect on the FRET signal, as reported previously^82,83^. Extracellular solution osmolality was 295–305 mOsm, measured with the Osmomat 030 freezing point osmometer (Gonotech GmbH, Germany).

In experiments with Epac1-camps, single-exponential increases to maximum functions (*F* = *F*_0_ + *c* × (1 − exp(−*t*/*τ*))) were fitted to the FRET ratio signals using SigmaPlot. The time constant (*τ*) and amplitude changes in the FRET ratio signal (ΔFRET [%]) were determined from the fitted curves. *F* is the FRET ratio signal at time *t*, *F*_0_ is the baseline FRET ratio signal, *c* is the FRET ratio signal amplitude of *F* − *F*_0_, and *τ* is the time constant of the individual exponential component. In experiments with Laconic, the maximal (initial) rates of the FRET ratio signal increase (ΔFRET/Δ*t*) were calculated as the slope of the linear regression function (ΔFRET [%] = slope [%/min] × Δ*t* [min]) fitting the initial FRET ratio signal increase. In these experiments, changes in the FRET ratio signal (ΔFRET [%]) were calculated by subtracting the mean maximal FRET ratio signals from the mean baseline FRET ratio signals.

Unless stated otherwise, the Student’s t-test was performed to determine statistical significance between the experimental groups; *P* < 0.05 was considered significant.

### Fluo-4 AM measurements of cytosolic Ca^2+^ and data analysis

Astrocytes expressing RFP-TDP-43^wt^ or RFP-TDP-43^208–414^ were incubated for 30 min at room temperature in medium containing 2 µM Fluo-4 AM dye (Molecular Probes, Invitrogen) and then transferred to dye-free medium for at least 30 min before the experiments to allow for cleavage of the AM ester group. The cells were excited with an Argon-ion laser at 488 nm, and time-lapse images were obtained every 3 s with an inverted Zeiss LSM780 confocal microscope and an 20x objective. Emission light was acquired with a 505–530-nm band-pass emission filter. Fluo-4 AM–labeled astrocytes were after 100 s baseline stimulated with 100 μM NA. Total recording time was ~400 s (135 frames). In individual cells, Fluo-4 AM intensity was quantified within a region of interest and expressed as the relative change in fluorescence: ΔF/F_0_ = (F − F_0_)/F_0_, where F_0_ denotes the baseline fluorescence level after subtraction of background fluorescence. The peak and cumulative increase in Fluo-4 AM ΔF/F_0_ were determined using Microsoft Excel.

Mann-Whitney U test was performed to determine statistical significance between the experimental groups. *P* < 0.05 was considered significant.

## Supplementary information

Supplementary material.

## Data Availability

All data generated or analyzed during this study are included in this published article and its Supplementary Information files or are available from the corresponding author on reasonable request.
