# Supplementary material for: Astrocytes with TDP-43 inclusions exhibit reduced noradrenergic cAMP and Ca2+ signaling and dysregulated cell metabolism
Source: Sci Rep. 2020 Apr 7;10:6003. doi: 10.1038/s41598-020-62864-5 (PMC7138839; doi:10.1038/s41598-020-62864-5)
Supplement: Supplementary file 1 — Supplementary material. [file 41598_2020_62864_MOESM1_ESM.docx]

# Supplementary Material_Scientific Reports

**Astrocytes with TDP-43 inclusions exhibit reduced noradrenergic cAMP and Ca^2+^ signaling and dysregulated cell metabolism**

Jelena Velebit^1,†^, Anemari Horvat^1,2,†^, Tina Smolič^2^, Sonja Prpar Mihevc^3^, Boris Rogelj^3,4,5^, Robert Zorec^1,2^, Nina Vardjan^1,2,^*

^1^Laboratory of Cell Engineering, Celica Biomedical, 1000 Ljubljana, Slovenia

^2^Laboratory of Neuroendocrinology – Molecular Cell Physiology, Institute of Pathophysiology, Faculty of Medicine, University of Ljubljana, 1000 Ljubljana, Slovenia

^3^Department of Biotechnology, Jožef Stefan Institute, 1000 Ljubljana, Slovenia

^4^Biomedical Research Institute BRIS, 1000 Ljubljana, Slovenia

^5^Faculty of Chemistry and Chemical Technology, University of Ljubljana, 1000 Ljubljana, Slovenia

*Correspondence: [nina.vardjan@mf.uni-lj.si](mailto:nina.vardjan@mf.uni-lj.si)

^†^Jelena Velebit and Anemari Horvat contributed equally to this work.


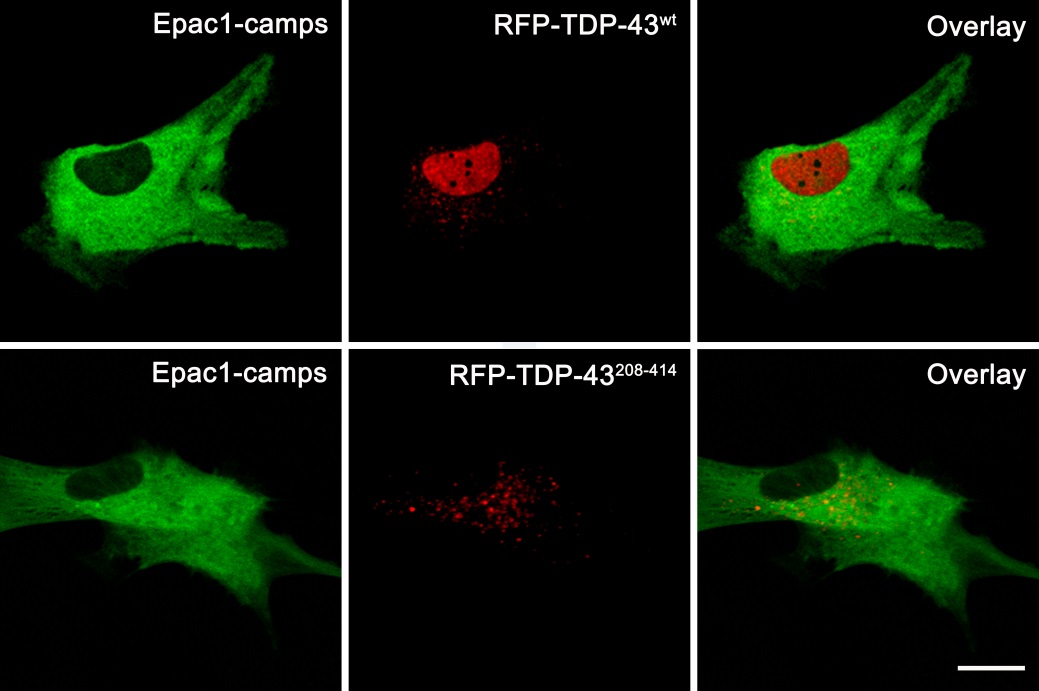


**Fig. S1** Co-expression of Epac1-camps and RFP-TDP-43^wt^ or RFP-TDP-43^208–414^ construct in primary cultures of rat astrocytes. Representative fluorescence images of astrocytes 30 h after co-transfection with Epac1-camps (green) and RFP-tagged TDP-43^wt^ (RFP-TDP-43^wt^; upper panels) or TDP-43^208–414^ (RFP-TDP-43^208–414^; lower panels) pDNA constructs (red). Note the preferential nuclear and cytoplasmic distribution for RFP-TDP-43^wt^ and RFP-TDP-43^208–414^ red fluorescence signals, respectively. Scale bar, 20 µm.


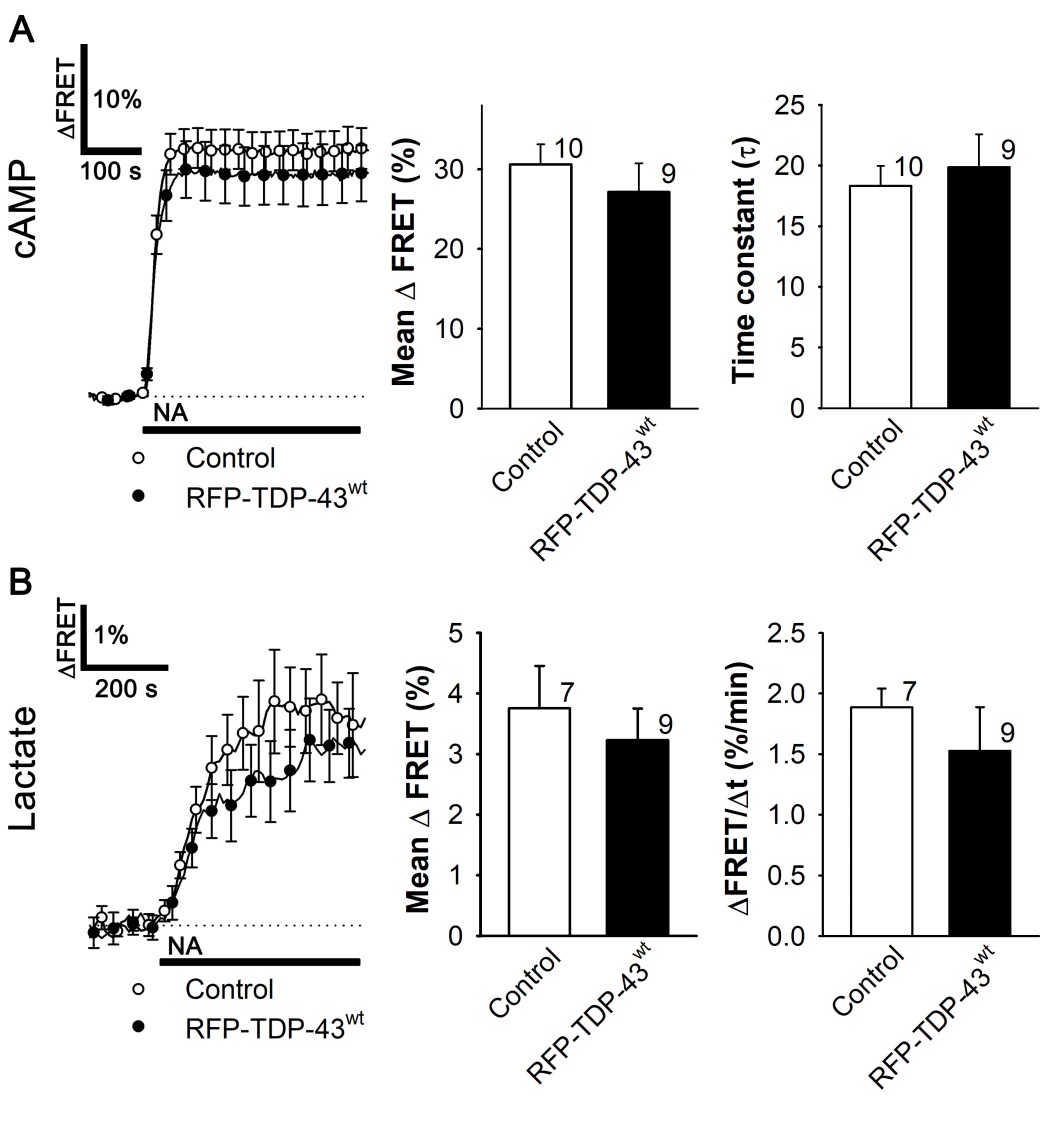


**Fig. S2** Noradrenaline-induced increases in [cAMP]_i_ and [lactate]_i_ are similar in control, non-transfected, and RFP-TDP-43^wt^-expressing astrocytes, respectively. **A, B** Left panels: average time-dependent changes in the Epac1-camps (**A**) and Laconic (**B**) FRET signal (ΔFRET) after the addition of NA (100 μM; black lines) in control non-transfected (white circles) and astrocytes expressing RFP-TDP-43wt (black circles). Data are expressed as percentages of the inverse FRET signal (CFP/YFP and mTFP/Venus for Epac1-camps and Laconic, respectively) relative to the baseline FRET signal. Each data point represents the mean ± SEM. Middle panels: Mean changes in the Epac1-camps (A) and Laconic (B) FRET signal (mean ΔFRET); right panels: mean time constants (τ) and mean initial rates of the FRET signal increase (ΔFRET/Δt) for Epac1-camps (**A**) and Laconic (**B**), respectively, after the addition of NA in control, non-transfected (white bars), and RFP-TDP-43^wt^-expressing (black bars) astrocytes. Numbers adjacent to the error bars depict the number of cells analysed. Data are presented as means ± SEM and acquired from at least two different animals (one cell was recorded per coverslip).
